# Major Characteristics of Severity and Mortality in Diabetic Patients With COVID-19 and Establishment of Severity Risk Score

**DOI:** 10.3389/fmed.2021.655604

**Published:** 2021-06-07

**Authors:** Yu-Feng Xiao, Jia-Lin He, Yu Xu, Xi Liu, Hui Lin, Qi Li, Zhi Xu, Ming-Dong Hu, Xiao-Bao Ren, Cheng Zhang, Wen-Jing Zhang, Wei Duan, Yong-Feng Tian, Ping Li, Hao Wu, Cai-Ping Song, En Liu, Shi-Ming Yang

**Affiliations:** ^1^Department of Gastroenterology, Xinqiao Hospital, The Army Medical University, Chongqing, China; ^2^Huo-Shen-Shan Hospital, Wuhan, China; ^3^The Medical Team of the Army Medical University, Jin-Yin-Tan Hospital, Wuhan, China; ^4^Department of Respiratory and Critical Care Medicine, Xinqiao Hospital, The Army Medical University, Chongqing, China; ^5^Taikang Tongji Hospital, Wuhan, China; ^6^Department of Emergency, Xinan Hospital, The Army Medical University, Chongqing, China; ^7^Department of Hematology, Xinqiao Hospital, The Army Medical University, Chongqing, China; ^8^Department of Neurology, Xinqiao Hospital, The Army Medical University, Chongqing, China; ^9^Department of Endocrinology, Xinqiao Hospital, The Army Medical University, Chongqing, China; ^10^Department of Cardiology, Xinqiao Hospital, The Army Medical University, Chongqing, China; ^11^Xinqiao Hospital, The Army Medical University, Chongqing, China

**Keywords:** diabetes, COVID-19, severity, mortality, risk score

## Abstract

**Objectives:** Diabetes is a risk factor for poor COVID-19 prognosis. The analysis of related prognostic factors in diabetic patients with COVID-19 would be helpful for further treatment of such patients.

**Methods:** This retrospective study involved 3623 patients with COVID-19 (325 with diabetes). Clinical characteristics and laboratory tests were collected and compared between the diabetic group and the non-diabetic group. Binary logistic regression analysis was applied to explore risk factors associated in diabetic patients with COVID-19. A prediction model was built based on these risk factors.

**Results:** The risk factors for higher mortality in diabetic patients with COVID-19 were dyspnea, lung disease, cardiovascular diseases, neutrophil, PLT count, and CKMB. Similarly, dyspnea, cardiovascular diseases, neutrophil, PLT count, and CKMB were risk factors related to the severity of diabetes with COVID-19. Based on these factors, a risk score was built to predict the severity of disease in diabetic patients with COVID-19. Patients with a score of 7 or higher had an odds ratio of 7.616.

**Conclusions:** Dyspnea is a critical clinical manifestation that is closely related to the severity of disease in diabetic patients with COVID-19. Attention should also be paid to the neutrophil, PLT count and CKMB levels after admission.

## Highlights

- Dyspnea is a critical clinical manifestation that is closely related to the severity of disease in diabetic patients with COVID-19. Attention should also be paid to the neutrophil, PLT count and CKMB levels after admission.
- Different from previous study, our study found that CRP did not predict the severity and death of diabetic patients after infected with COVID-19.
- Based on these factors, a risk score was built to predict the severity of disease in diabetic patients with COVID-19. Patients with a score of 7 or higher had an odds ratio of 7.616.

## Introduction

At the end of 2019, a newly identified virus, termed COVID-19, began spreading rapidly through China and the rest of the world, which has become a global catastrophe. As of August 1, 2020, at least 17 million patients worldwide have been diagnosed with COVID-19, with more than 670,000 deaths, and the global epidemic has not stopped yet. COVID-19 is highly infectious, and many patients worsen very quickly after infection. Acute respiratory distress syndrome (ARDS), multiple organ dysfunction syndrome (MODS) and septic shock are commonly found in severe cases (1).

Diabetes has been reported as a frequent comorbidity in COVID-19 patients (2). Recent studies have shown that diabetic patients with COVID-19 may have a more than 50% higher rate of experiencing a fatal outcome than those who do not have diabetes (3). Guo et al. (4) reported that diabetes should be considered a risk factor for a rapid progression and poor prognosis of COVID-19. Yan et al. (5) found that of 193 patients with severe COVID-19, 48 (24.9%) had diabetes, and diabetics had a higher risk of death compared with those who did not have diabetes. Chen and colleagues demonstrated that older diabetic patients with COVID-19 were at increased risk of death (6). Interestingly, a recent study showed that diabetic patients with well-controlled blood glucose had markedly lower mortality compared to those with poorly controlled blood glucose in diabetics with COVID-19(4). However, these recent studies did not clarify why diabetic patients with COVID-19 had different outcomes or what factors contribute to the increased severity and risk of death in diabetic patients with COVID-19. Few studies integrated multiple risk factors into the risk prediction score which was capable of accurately stratifying diabetic patients with COVID-19 into different risk groups on the basis of clinical data. It would be undoubtedly of great significance for clinical work if a clinician could prejudge which patients have a higher risk of severe disease and death at the time at which the patient begins clinical treatment.

Based on the above expectations, a retrospective multicenter study of a cohort of 3623 patients diagnosed with COVID-19 from three different hospitals in Hubei, China, was performed. The basic information, clinical manifestations and laboratory tests at admission were collected into the database of study indicators, and the factors leading to different outcomes in diabetic patients after infection with COVID-19 were evaluated. Based on these clinical data, we sought to develop a risk stratification score capable of identifying severity of diabetic patients with COVID-19 using clinical data to facilitate the target of rapid evaluation of patients' risk of critical and death, providing guidance for subsequent treatment.

## Methods

### Study Design and Participants

This retrospective study included 3,623 patients who were admitted to three hospitals (HuoShenShan Hospital, Jinyintan Hospital and Taikang Tongji Hospital) in Wuhan, Hubei Province, China. There were 2271 COVID-19 patients admitted to HSS Hospital from Febryary 4, 2020 to March 31, 2020; they were retrospectively screened and followed until April 15, 2020 or until HSS Hospital closed. A total of 152 patients were excluded due to duplicate data, and 2119 patients were ultimately included. Ninety-five COVID-19 patients were admitted to Jinyintan Hospital from January 26, 2020 to February 1, 2020; two patients were excluded due to missing data, and one patient was excluded due to death upon arrival. A total of 1412 COVID-19 patients from Taikang Tongji Hospital, admitted from February 19, 2020 to April 2, 2020, were also included in this study. No patients were excluded from this cohort. These three hospitals were class A tertiary comprehensive hospitals designated to treat patients with COVID-19. Most patients included in this study were local residents. Patients diagnosed according to the World Health Organization (WHO) interim guidance for COVID-19 were included in the study. Diabetes was ascertained through previous medical records or self-reported diagnosis confirmed by clinicians. Diabetes was diagnosed according to WHO diagnostic criteria: blood glucose (>11.1 mmol/L) (200 mg/dl) at any time of the day, fasting blood glucose (>7.0 mmol/L) (126 mg/dl), or oral glucose tolerance test (>11.1 mmol/L) (200 mg/dl) at 2 h. We did not further classify diabetes in the 325 diabetic patients.

### Ethics Statement

The study was approved by the ethics committee of Xinqiao Hospital (2020-yd073-01) with written informed consent waived due to the retrospective nature of the study. This study was carried out according to the Strengthening the Reporting of Observational Studies in Epidemiology (STROBE) reporting guidelines.

### Inclusion and Exclusion Criteria

Inclusion criteria: Patients were diagnosed with COVID-19, according to the standard WHO (World Health Organization) diagnostic criteria.

Exclusion criteria: (1) Suspected patients were diagnosed and excluded from COVID-19 infection; (2) patients had died upon arrival without treatment; (3) missing data.

### Data Collection

Patients enrolled in this study were divided into two groups according to the diagnosis of diabetes. The demographic data (such as sex and age), clinical symptoms (such as fever, cough, sputum, dyspnea(According to the “New Coronavirus Infection Pneumonia Diagnosis and Treatment Plan,” released by the National Health and Health Council of China, dyspnea is defined as respiratory distress (frequency >30 times/min), resting state oxygen saturation is <93%), chest tightness, hemoptysis, fatigue, nausea, abdominalgia, diarrhea, anorexia), vital signs (body temperature, breathing rate, heart rate, blood pressure) and basic laboratory test (which were carried out in approved labs with internal quality controls) results at admission were reviewed and extracted by experienced clinicians using a standardized data collection form. The COVID-19 severity grading (mild, moderate, severe, or critical) was defined according to the Diagnosis and Treatment Plan for COVID-19 issued by the NHC of China.

### Sample Size Evaluation

In this study, 325 cases of diabetic patients infected with COVID-19, who met the inclusion and exclusion criteria, were included for analysis. Among these patients, severe group and the mild group contained 115 and 210 cases, respectively. According to the *p* value, the final 10 factors were selected for logistic regression analysis. Finally, based on the statistical results, five factors were used as the scoring indicators, which was in conformity with the principle of beyond the 10 events per variable (EPV) rule of thumb (Richard, DR, et al. BMJ. 2020). The minimum sample size of each group is 5^*^10 = 50.

### Statistical Analysis

Continuous variables were expressed as the median (interquartile range [IQR]) or mean ± SD, and categorical variables were presented as *n* (%). The differences between groups were compared by using the Mann-Whitney U test, *t* test, χ2 test, or Fisher's exact test, as appropriate. The determination of odds ratios (ORs) and 95% CIs for factors associated with clinical outcomes were analyzed by binary logistic regression analysis. A prediction model was built based on the results of binary logistic regression analysis. Each predictor in the final model was weighted based on the estimated coefficient. An ROC curve was created by using the risk scores to evaluate the sensitivity and specificity. The Hosmer–Lemeshow goodness-of-fit test was applied to assess the risk score model calibration. Statistical analyses were performed using IBM SPSS 23 statistics software (SPSS Inc., Chicago, IL, United States) and R software (version 3.4, R Foundation, Vienna, Austria. www. R-project. org). All *p*-values were two-sided, and *P* < 0.05 was considered statistically significant.

## Results

### Clinical Characteristics of Patients With COVID-19 Upon Admission

**Figure 1:**
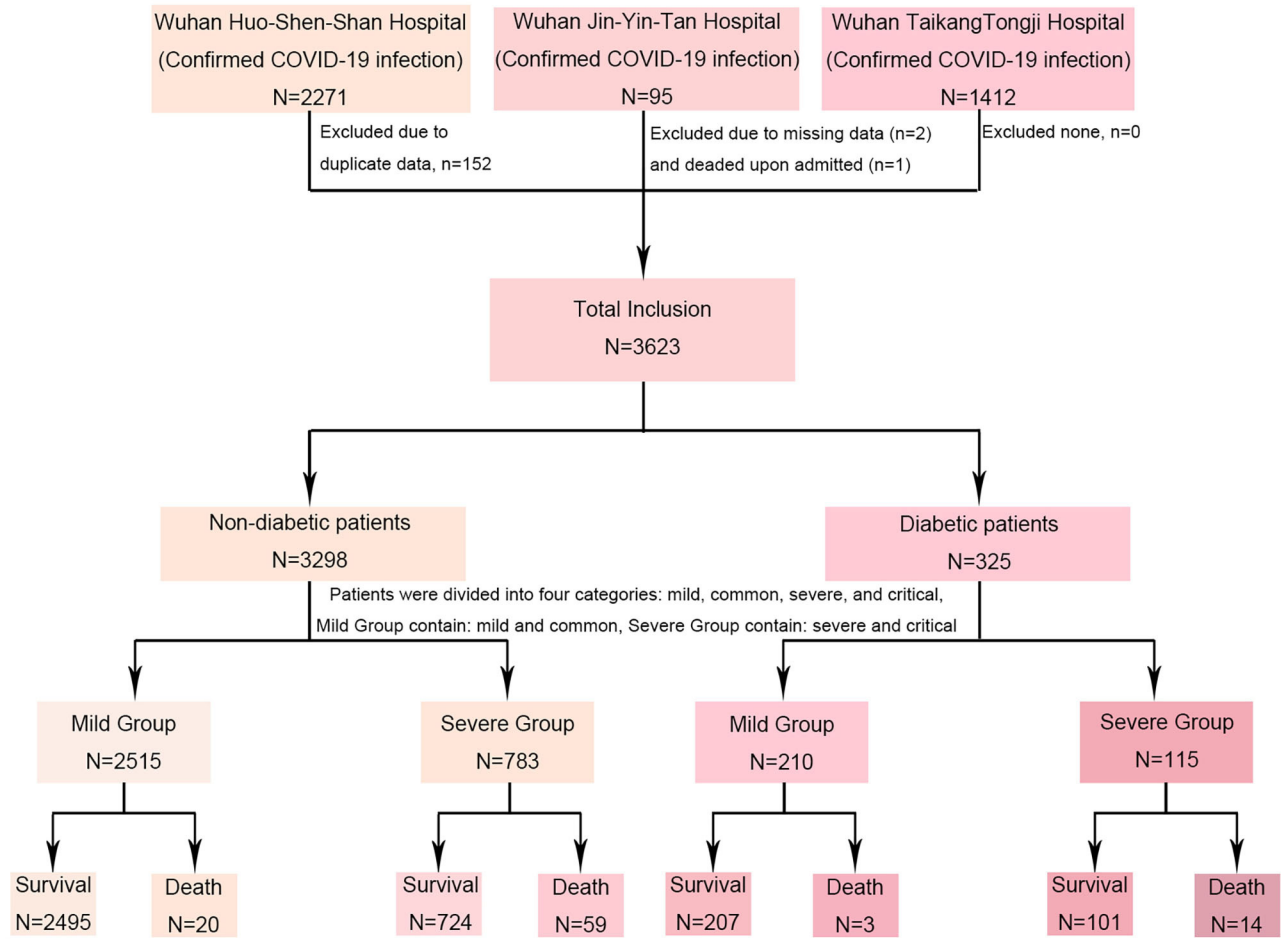
Enrollment flow chart of study population.

Clinical characteristics of the 3,623 patients with confirmed COVID-19 from three hospitals in Wuhan, Hubei, China were collected (Figure 1); this cohort included 325 patients with pre-existing diabetes and 3,298 non-diabetic patients (Table 1). The median ages were 66 (58–72) and 61 (49–69) in the diabetic and non-diabetic groups, respectively. There were no significant differences between the diabetic and non-diabetic groups in terms of clinical basic vital signs, such as body temperature (37.69 vs. 37.61°C), respiratory rate (20/min vs. 20/min), blood pressure (133/80 vs. 130/80 mmHg) or pulse (86/min vs. 84/min). The diabetic group exhibited significantly higher incidence rates of dyspnea (29.85 vs. 23.65%, *p* = 0.013) than the non-diabetic groups, while no significant difference was found in the prevalence of cough (69.54 vs. 64.43%), expectoration (13.54 vs. 12.25%), chest tightness (20.92 vs. 20.16%), or hemoptysis (0 vs. 0.24%) between the two groups. It seems that diabetic patients exhibited a higher incidence of loss of appetite (31.38 vs. 25.86%, *p* = 0.031) than non-diabetic patients, and no significant differences were observed in other clinical manifestations of the digestive tract, such as vomiting (2.15 vs. 2.33%), abdominal pain (0.31 vs. 1.33%), and diarrhea (4 vs. 5.25%), between these two groups. Interestingly, pre-existing cardiovascular diseases (including hypertension and coronary heart disease) had a higher frequency in the diabetic group than in the non-diabetic group (55.38 vs. 23.53%, *p* < 0.001), similar to a previous report.

**Table 1:** Basic characteristics of diabetic and non-diabetic patients with COVID-19 (*N* = 3,623).

	**Total**	**Non-diabetic**	**Diabetic**	***P-*value**
	**(*N* = 3,623)**	**(*n* = 3,298)**	**(*n* = 325)**	
Male, *n* (%)	1,766(48.74)	1,589(48.18)	177(54.46)	0.031
Female, *n* (%)	1,857(51.26)	1,709(51.82)	148(45.54)	0.031
Age, median (IQR)	61(50–69)	61(49~69)	66(58–72)	0.000
Body temperature, Mean ± SD, °C	37.62 ± 1.04	37.61 ± 1.04	37.69 ± 1.04	0.203
Fever, *n* (%)	2,319(64.01)	2,099(63.64)	220(67.69)	0.147
Respiratory rate, *n*/min	20(18–21)	20(18~21)	20(19~22)	0.007
Pulse, *n*/min	84(78–96)	84(78~95)	86(78~96)	0.066
SBP, median (IQR), mmHg	130(120–140)	130(120~140)	133(124~143)	0.000
DBP, median (IQR), mmHg	80(73–88)	80(73~88)	80(74.5~89)	0.279
Fatigue, *n* (%)	1,799(49.65)	1,620(49.12)	179(55.08)	0.040
Duration of first symptom, day (IQR)	20.5(14–30)	21.0(14–30)	20.0(13–30)	0.793
**Respiratory symptoms**				
Cough, *n* (%)	2,351(64.89)	2,125(64.43)	226(69.54)	0.066
Expectoration, *n* (%)	448(12.37)	404(12.25)	44(13.54)	0.501
Dyspnea, *n* (%)	877(24.21)	780(23.65)	97(29.85)	0.013
Chest tightness, *n* (%)	733(20.23)	665(20.16)	68(20.92)	0.745
Hemoptysis, *n* (%)	8(0.22)	8(0.24)	0(0)	1.000
**Digestive tract symptoms**				
Vomiting, *n* (%)	84(2.32)	77(2.33)	7(2.15)	0.836
Abdominal pain, *n* (%)	45(1.24)	44(1.33)	1(0.31)	0.175
Diarrhea, *n* (%)	186(5.13)	173(5.25)	13(4.00)	0.332
Anorexia, *n* (%)	955(26.36)	853(25.86)	102(31.38)	0.031
**Past medical history**				
Cardiovascular disease^a^, *n* (%)	956(26.39)	776(23.53)	180(55.38)	0.000
Lung diseases^b^, *n* (%)	159(4.39)	147(4.46)	12(3.69)	0.521
Liver disease^c^, *n* (%)	107(2.95)	99(3.00)	8(2.46)	0.583
WBC, median (IQR), 10^∧^9/L	5.80(4.80~7.00)	5.80(4.8~6.98)	5.80(4.8~7.2)	0.525
Neutrophil, median (IQR),10^∧^9/L	3.48(2.68~4.54)	3.45(2.66~4.52)	3.68(2.86~5.08)	0.007
Lymphocyte, median (IQR), 10^∧^9/L	1.52(1.12~1.88)	1.53(1.14~1.89)	1.38(0.99~1.74)	0.000
Proportion of neutral lymph, median (IQR)	2.31(1.65~3.33)	2.26(1.63~3.26)	2.67(1.84~4.3)	0.000
HGB, median (IQR), g/L	122(111~133)	122(111~133)	120(108.5~131)	0.044
PLT, median (IQR), 10^∧^9/L	225(183~272)	226(184~272)	215(176~269)	0.058
Bilirubin, median (IQR), umol/L	10.28(7.80~13.25)	10.30(7.90~13.25)	9.50(6.88~12.98)	0.001
ALT, median (IQR), IU/L	24.40(15.10~37.00)	24.70(15.20~37.83)	20.10(13.95~33.15)	0.000
AST, median (IQR), IU/L	22.80(17.1~37.35)	23.10(17.30~37.35)	19.10(14.50~27.98)	0.000
ALB, median (IQR), g/L	37.85(34.72~40.21)	37.85(34.84~40.30)	36.96(33.50~39.55)	0.000
CRP, median (IQR), mg/L	1.99(0.50–7.00)	1.81(0.50~6.42)	4.64(1.21~17.05)	0.000
CREA, median (IQR), umol/L	62.40(51.94–75.70)	62.10(51.58~75.23)	64.90(54.40~79.60)	0.007
CKMB, median (IQR), ng/mL	8.90(6.70–11.63)	8.89(6.63~11.42)	9.60(7.60~13.95)	0.000
MuLBSTA score, median (IQR)	5(7–9)	5(7–9)	9(7–11)	0.000
**Diagnosis type**				
Mild and Common, *n* (%)	2,725(75.21)	2,515(76.26)	210(64.62)	0.000
Severe and Critical, *n* (%)	898(24.79)	783(23.74)	115(35.38)	
Death, *n* (%)	96(2.65)	79(2.40)	17(5.23)	0.002

*SBP, systolic blood pressure; DBP, diastolic blood pressure; WBC, white blood cell; HGB, hemoglobin; PLT, platelet; ALB, albumin; ALT, alanine aminotransferase; AST, aspartate aminotransferase; CRP, C-reactive protein; CREA, creatinine; CKMB, creatine phosphokinase isoenzyme*.

a *Cardiovascular disease includes coronary heart disease and hypertension and etc*.

b *Lung disease includes chronic bronchitis, COPD, tuberculosis and lung cancer and etc*.

c *Liver disease includes hepatitis B, hepatitis C, fatty liver, cirrhosis, liver cancer, hepatitis A, hepatic hemangioma, schistosomiasis liver disease and etc*.

The two groups showed greater differences in laboratory test results. There was no significant difference in WBC (white blood cell) count between the diabetic and non-diabetic groups (5.8 × 10^∧^9/L vs. 5.8 × 10^∧^9/L); however, according to our findings, patients in the diabetic group had higher neutrophil (3.68 × 10^∧^9/L vs. 3.45 × 10^∧^9/L, *p* = 0.007) and lower lymphocyte (1.38 × 10^∧^9/L vs. 1.53 × 10^∧^9/L, *p* < 0.001). In addition, CRP (C-reactive protein) also showed higher levels in the diabetic group than in the non-diabetic group (4.64 vs. 1.81 mg/L, *p* < 0.001). These results may indicate that diabetic patients have a higher inflammatory response base, and their autoimmune function is different from that of non-diabetic patients. Surprisingly, the liver function indexes [ALT (20.1 vs. 24.7 IU/L, *p* < 0.001), AST (19.1 vs. 23.1 IU/L, *p* < 0.001) and total bilirubin (9.5 vs. 10.28 umol/L, *p* = 0.001)] levels of diabetic patients were better than those of non-diabetic patients. At the same time, creatinine (64.9 vs. 62.1 umol/L, *p* = 0.007) and creatine kinase-MB (CKMB) (9.6 vs. 8.89 ng/mL, *p* < 0.001) were higher in the diabetic group than in the non-diabetic group. Furthermore, diabetic patients gained a higher MuLBSTA score than non-diabetic patients (9 vs. 5, *p* < 0.001). Importantly, we also found that the diabetic group also showed a higher rate of severe cases (35.38 vs. 23.74%, *p* < 0.001) and deaths (5.23 vs. 2.40%, *p* = 0.002) compared with the non-diabetic group, suggesting that diabetic patients may need more intensive care during their in-hospital treatment.

### Different Clinical Characteristics Risk Factors for COVID-19 Severity Between Diabetic and Non-diabetic Patients

Based on previous studies and our results, diabetic patients have a significantly higher probability of severe disease and death after infection with COVID-19 than non-diabetic patients. However, the factors that could lead to this worse result in the diabetic group have not been confirmed by relevant studies, so we further analyzed the diabetic patients infected with COVID-19. Diabetic patients were divided into the mild group (MG, contain mild and common type) and severe group (SG, contain severe and critical type) according to the Clinical Characteristics of Coronavirus Disease 2019 in China (1), and differences between the two groups were further discussed (Table 2).

**Table 2:** Univariate analysis of severity-related factors in diabetic patients with COVID-19, (*N* = 325).

	**Total**	**MG**	**SG**	***P-*value**
	**(N = 325)**	**(*N* = 210)**	**(*N* = 115)**	
Male, *n* (%)	177(54.46)	107(50.95)	70(60.87)	0.086
Female, *n* (%)	148(45.54)	103(49.05)	45(39.13)	0.086
Age, median (IQR)	66(58~72)	65(57~72)	68(60~74)	0.039
Body temperature, mean ± SD, °C	37.69 ± 1.04	37.73 ± 1.02	37.61 ± 1.08	0.345
Fever, *n* (%)	220(67.69)	147(70.00)	73(63.48)	0.229
Respiratory rate, *n*/min	20(19~22)	20(19~21)	20(19~22)	0.004
Pulse, *n*/min	86(78~96)	85(78~94)	89(80~100)	0.012
SBP, median (IQR), mmHg	133(124~143)	132(124~143)	133(125~144)	0.547
DBP, median (IQR), mmHg	80(74.5~89)	80(75~89)	80(73~88)	0.577
Fatigue, *n* (%)	179(55.08)	121(57.62)	58(50.43)	0.213
Duration of first symptom, day (IQR)	20(13~30)	20(12.75~30)	23(14~30)	0.313
**Respiratory symptoms**				
Cough, *n* (%)	226(69.54)	143(68.10)	83(72.17)	0.445
Expectoration, *n* (%)	44(13.54)	26(12.38)	18(15.65)	0.410
Dyspnea, *n* (%)	97(29.85)	48(22.86)	49(42.61)	0.000
Chest tightness, *n* (%)	68(20.92)	39(18.57)	29(25.22)	0.159
Hemoptysis, *n* (%)	0(0)	0(0)	0(0)	
**Digestive tract symptoms**				
Vomiting, *n* (%)	7(2.15)	5(2.38)	2(1.74)	1.000
Abdominal pain, *n* (%)	1(0.31)	1(0.48)	0(0.00)	1.000
Diarrhea, *n* (%)	13(4.00)	8(3.81)	5(4.35)	0.776
Anorexia, *n* (%)	102(31.38)	62(29.52)	40(34.78)	0.329
**Past medical history**				
Cardiovascular disease^a^, *n* (%)	180(55.38)	104(49.52)	76(66.09)	0.004
Lung diseases^b^, *n* (%)	12(3.69)	7(3.33)	5(4.35)	0.643
Liver disease^c^, *n* (%)	8(2.46)	6(2.86)	2(1.74)	0.717
WBC, 10^∧^9/L	5.80(4.80~7.20)	5.51(4.70~6.80)	6.40(5.00~8.18)	0.001
Neutrophil, 10^∧^9/L	3.68(2.86~5.08)	3.50(2.79~4.37)	4.15(3.20~6.30)	0.000
Lymphocyte, 10^∧^9/L	1.38(0.99~1.74)	1.41(1.05~1.75)	1.33(0.77~1.72)	0.058
Proportion of neutral lymph	2.67(1.84~4.30)	2.54(1.72~3.32)	3.03(1.98~6.21)	0.000
HGB, g/L	120(108.50~131.00)	120(111.75~131)	120(103.5~132)	0.159
PLT, 10^∧^9/L	215(176~269)	221.5(184~274.5)	205(157~255)	0.014
Bilirubin, umol/L	9.50(6.88~12.98)	9.35(6.90~12.76)	10.30(6.40~13.50)	0.352
ALT, IU/L	20.10(13.95~33.15)	21.70(14.5~32.88)	18.00(12.30~34.40)	0.047
AST, IU/L	19.10(14.50~27.98)	19.60(15.38~27.96)	18.50(13.70~28.90)	0.214
ALB, g/L	36.96(33.50~39.55)	37.40(34.39~39.83)	35.90(31.80~38.90)	0.002
CRP, mg/L	4.64(1.21~17.05)	3.18(0.88~12.30)	7.76(1.86~34.09)	0.000
CREA, umol/L	64.90(54.40~79.60)	63.49(54.55~77.63)	66.80(53.90~81.60)	0.315
CKMB, ng/mL	9.60(7.60~13.95)	9.20(7.38~12.23)	11.31(7.80~21.80)	0.003
MuLBSTA score	9(7~11)	7(5~9)	9(8~13)	0.000

a *Cardiovascular disease includes coronary heart disease and hypertension and etc*.

b *Lung disease includes chronic bronchitis, COPD, tuberculosis and lung cancer and etc*.

c *Liver disease includes hepatitis B, hepatitis C, fatty liver, cirrhosis, liver cancer, hepatitis A, hepatic hemangioma, schistosomiasis liver disease and etc*.

The median ages were 68 (60–74) and 65 (57–72) in the SG and MG, respectively. The MG exhibited a quicker pulse than the SG [89/min (80–100) vs. 85/min (78–94), *p* = 0.012]. There was no significant difference between the two groups in terms of body temperature, fever, respiratory rate, blood pressure, fatigue or duration of first symptoms. In addition, we also found that the SG showed a higher rate of dyspnea than the MG (42.61 vs. 22.86%, *p* < 0.001), while no significant difference was found in terms of cough (72.17 vs. 68.1%), expectoration (15.65 vs. 12.38%), chest tightness (25.22 vs. 18.57%), or hemoptysis (0 vs. 0%) between the two groups. There was no difference in the incidence of gastrointestinal symptoms between the two groups. Pre-existing cardiovascular diseases were more frequent in the SG than in the MG (66.09 vs. 49.52%, *p* = 0.004). Moreover, the SG showed more severe inflammatory markers of infection than the MG, such as WBC count (6.40 × 10^∧^9/L vs. 5.51 × 10^∧^9/L, *p* = 0.001), neutrophil (4.15 × 10^∧^9/L vs. 3.50 × 10^∧^9/L, *p* < 0.001), and CRP (7.76 vs. 3.18 mg/L, *p* < 0.001). The SG also showed a higher level of CKMB than the MG (11.31 vs. 9.2 ng/mL, *p* = 0.003).

**Figure 2:**
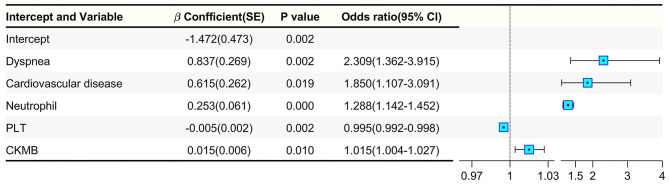
Binary logistic regression analysis of severity-related factors in diabetic patients with COVID-19.

To further explore the risk factors associated with the severe progression of diabetic patients infected with COVID-19, binary logistic regression analysis was applied (Figure 2). We found that dyspnea (*p* = 0.002, OR = 2.309), cardiovascular diseases (*p* = 0.019, OR = 1.850), neutrophil (*p* < 0.001, OR = 1.288), PLT count (*p* = 0.002, OR = 0.995), and CKMB (*p* = 0.010, OR = 1.015) were more common in the SG, suggesting that these five factors may strongly related with the severity of diabetic patients when infected with COVID-19. In addition, we also analyzed the risk factors in the non-diabetic group (Supplementary Tables 1, 2). Interestingly, the risk factors leading to COVID-19 severity of the non-diabetic groups were different from those of the diabetic group. Sex (*p* = 0.019, OR = 1.244), age (*p* < 0.001, OR = 1.040), dyspnea (*p* < 0.001, OR = 1.752), cardiovascular diseases (*p* = 0.009, OR = 1.298), respiratory rate (*p* < 0.001, OR = 1.028), WBC (*p* = 0.022, OR = 1.037), HGB (*p* < 0.001, OR = 0.987), ALB (*p* = 0.001, OR = 0.966) and CRP (*p* < 0.001, OR = 1.007) were related to COVID-19 severity in the non-diabetic group. Although dyspnea and cardiovascular diseases were related to disease severity in both the diabetic group and non-diabetic group, dyspnea and cardiovascular diseases might bring a greater risk of exacerbation in diabetic patients than in non-diabetic patients.

### Risk Factors for Diabetic and Non-diabetic in-hospital Mortality

We have discussed the risk factors that contributed to the severity of COVID-19 in diabetic patients. Previously, we also found that diabetic patients had a higher risk of death after infection with COVID-19 than non-diabetic patients. What were the risk factors that contributed to the high in-hospital mortality of diabetic patients?

The data from the 325 hospitalized diabetic patients with COVID-19 were collected and analyzed (Table 3). Patients with diabetes were divided into a survival group (SurG, *n* = 308) and a deceased group (DG, *n* = 17) according to patient outcome. The median age was 77 (66.5–82) and 65 (58–72) years in the DG and SurG, respectively, and the age of the DG was older than that of SurG (*p* = 0.001). No significant differences were found in body temperature, fever, respiratory rate, pulse, blood pressure, fatigue symptoms, or duration of first symptom between the two groups. Similar to Table 2, we also found that the frequency of dyspnea in the DG (*n* = 13, 76.47%) was much higher than that in SurG (*n* = 84, 27.27%) (*p* < 0.001); however, there was no significant difference in the incidence of cough, expectoration, chest tightness, hemoptysis and gastrointestinal symptoms (including vomiting, abdominal pain, diarrhea, anorexia) between the two groups. Interestingly, although no significant difference was found in the prevalence of pre-existing cardiovascular disease between the SurG and DG, the DG had a higher rate of coronary heart disease (*n* = 5, 29.41%) compared with the SG (*n* = 29, 8.92%). Our data also showed that the DG had a higher frequency of pre-existing lung disease (*n* = 3, 17.65%) than the SG (*n* = 9, 2.92) (*p* = 0.002), while no significant difference was found in liver disease. Similar to previous findings, the DG showed a higher WBC count [8.5 × 10^∧^9/L (6.65–12) vs. 5.7 × 10^∧^9/L (4.78–7), *p* < 0.001] and neutrophil [7.53 × 10^∧^9/L (5.16–10.88) vs. 3.64 × 10^∧^9/L (2.81–4.69), *p* < 0.001] but a lower lymphocyte (0.69 × 10^∧^9/L vs. 1.41 × 10^∧^9/L, *p* = 0.001), HGB level (103.50 vs. 120.50 g/L, *p* = 0.021), and PLT count (168.00 × 10^∧^9/L vs. 216.50 × 10^∧^9/L, *p* = 0.027). Higher levels of CRP (57.79 vs. 4.30 mg/ml, *p* < 0.001), CKMB (22.60 vs. 9.40 ng/ml, *p* < 0.001) and creatinine (82.60 vs. 64.43 umol/L, *p* = 0.031) were also observed in the DG than in the SurG.

**Table 3:** Univariate analysis of death-related factors in diabetic patients with COVID-19 (*N* = 325).

	**Total**	**Survival**	**Death**	***P-*value**
	**(*N* = 325)**	**(*n* = 308)**	**(*n* = 17)**	
Male, *n* (%)	177(54.46)	165(53.57)	12(70.59)	0.710
Female, *n* (%)	148(45.54)	143(46.43)	5(29.41)	0.710
Age, median (IQR)	66(58~72)	65(58~72)	77(66~82)	0.001
Body temperature, Mean ± SD, °C	37.8 ± 1.04	37.8 ± 1.03	38.03 ± 1.17	0.170
Fever, *n* (%)	220(67.69)	208(67.53)	12(70.59)	0.793
Respiratory rate, *n*/min	20(19~22)	20(19~22)	20(19~22)	0.009
Pulse, *n*/min	86(78~96)	86(78~96)	86(78~96)	0.030
SBP, median (IQR), mmHg	133(124~143)	133(124~143)	133(124~143)	0.855
DBP, median (IQR), mmHg	80(74.5~89)	80(74.5~89)	80(74.5~89)	0.763
Fatigue, *n* (%)	179(55.08)	168(54.55)	11(64.71)	0.412
Duration of first symptom, day (IQR)	20(13~30)	20(13~30)	20(13~30)	0.165
**Respiratory symptoms**				
Cough, *n* (%)	226(69.54)	211(68.51)	15(88.24)	0.085
Expectoration, *n* (%)	43(13.23)	41(13.31)	3(17.65)	0.611
Dyspnea, *n* (%)	97(29.85)	84(27.27)	13(76.47)	0.000
Chest tightness, *n* (%)	68(20.92)	64(20.78)	4(23.53)	0.786
Hemoptysis, *n* (%)	0(0)	0(0)	0(0)	
**Digestive tract symptoms**
Vomiting, *n* (%)	7(2.15)	7(2.27)	0(0)	1.000
Abdominal pain, *n* (%)	1(0.31)	1(0.32)	0(0)	1.000
Diarrhea, *n* (%)	13(4.00)	13(4.22)	0(0)	1.000
Anorexia, *n* (%)	102(31.38)	95(30.84)	7(41.18)	0.371
**Past medical history**				
Cardiovascular disease^a^, *n* (%)	180(55.38)	170(55.19)	10(58.82)	0.770
Lung diseases^b^, *n* (%)	12(3.69)	9(2.92)	3(17.65)	0.002
Liver disease^c^, *n* (%)	8(2.46)	7(2.27)	1(5.88)	0.352
WBC, 10^∧^9/L	5.80(4.80~7.20)	5.70(4.78~7.00)	8.50(6.65~12.00)	0.000
Neutrophil, 10^∧^9/L	3.68(2.86~5.08)	3.64(2.81~4.69)	7.53(5.16~10.88)	0.000
Lymphocyte, 10^∧^9/L	1.38(0.99~1.74)	1.41(1.00~1.75)	0.69(0.36~1.28)	0.001
Proportion of neutral lymph	2.67 (1.84~4.30)	2.65 (1.80~3.80)	16.09(3.84~17.93)	0.000
HGB, g/L	120.00(108.50~131.00)	120.50(110.25~131.00)	103.50(87.00~128.00)	0.021
PLT, 10^∧^9/L	215.00(176.00~269.00)	216.50(178.13~269.50)	168.00(72.00~263.50)	0.027
Bilirubin, umol/L	9.50(6.88~12.98)	9.40(6.81~12.78)	12.60(7.70~22.40)	0.050
ALT, IU/L	20.10(13.95~33.15)	19.75(13.93~33.03)	24.90(15.55~41.40)	0.323
AST, IU/L	19.10(14.50~27.98)	18.83(14.43~26.85)	31.80(19.90~59.05)	0.001
ALB, g/L	36.96(33.50~39.55)	37.15(33.83~39.60)	32.00(27.10~37.25)	0.002
CRP, mg/L	4.64(1.21~17.05)	4.30(1.17~15.80)	57.79(7.14~169.28)	0.000
CREA, umol/L	64.90(54.40~79.60)	64.43(54.00~77.45)	82.60(56.40~157.30)	0.031
CKMB, ng/ml	9.60(7.60~13.95)	9.40(7.43~13.48)	22.60(10.40~44.23)	0.000
MuLBSTA Score	9(7~11)	9(7~10)	13(10~15)	0.000
**Diagnosis type**				
Mild and Common	210(64.62)	207(67.21)	3(17.65)	0.000
Severe and Critical	115(35.38)	101(32.79)	14(82.35)	

a *Cardiovascular disease includes coronary heart disease and hypertension and etc*.

b *Lung disease includes chronic bronchitis, COPD, tuberculosis and lung cancer and etc*.

c *Liver disease includes hepatitis B, hepatitis C, fatty liver, cirrhosis, liver cancer, hepatitis A, hepatic hemangioma, schistosomiasis liver disease and etc*.

**Figure 3:**
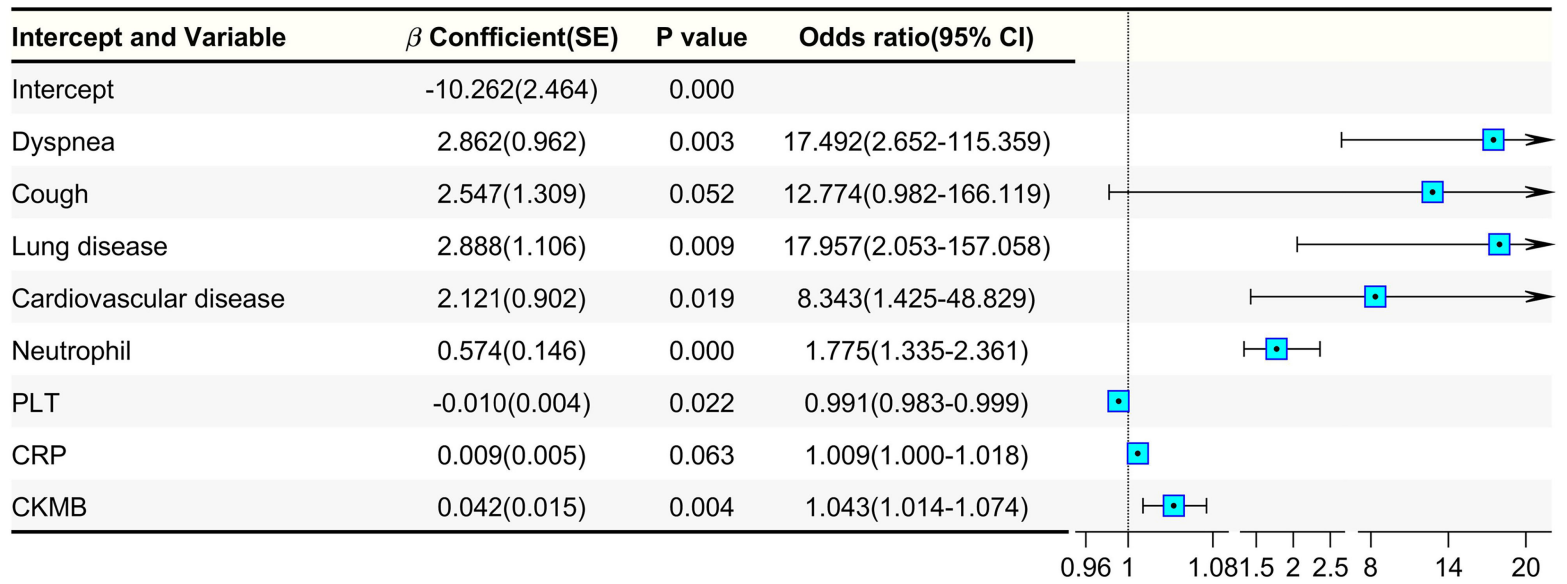
Binary logistic regression analysis of death-related factors in diabetic patients with COVID-19.

Binary logistic regression analysis was also applied to explore the risk factors associated with death in diabetic patients infected with COVID-19 (Figure 3). Dyspnea (*p* = 0.003, OR = 17.492), coronary heart disease (*p* = 0.019, OR = 8.343), neutrophil (*p* < 0.001, OR = 1.775), PLT count (*p* = 0.022, OR = 0.991), and CKMB (*p* = 0.004, OR = 1.014) were related to the risk of death among diabetic patients who were infected with COVID-19. Compared with the risk factors for non-diabetic patient death (Supplementary Tables 3, 4), we found that age (*p* = 0.002, OR = 1.037), dyspnea (*p* = 0.002, OR = 2.347), WBC count (*p* < 0.001, OR = 1.037), albumin (*p* < 0.001, OR = 0.987), PLT count (*p* = 0.001, OR = 0.966) and CRP (*p* < 0.001, OR = 1.007) were related to the risk of death in non-diabetic patients infected with COVID-19. Although no significant difference was found in CRP and cough in diabetic patients, these two indicators are extremely important for mortality risk prediction in non-diabetic patients. Regardless of diabetes mellitus, the independent risk factors for death included dyspnea, neutrophil, PLT count, and CRP. Previous studies suggested that CRP could be an independent risk factor for death in diabetic patients with COVID-19; however, according to our data, we believe that CRP may be an important risk factor for mortality risk in all patients with COVID-19, independent of diabetes mellitus status.

### Establishment of Risk Score of Diabetic Patients With COVID-19 (DPCR Score)

**Figure 4:**
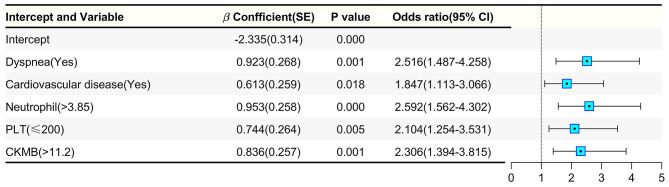
The weight coefficient was calculated by two classification.

Although many studies have reported that different risk factors, both clinical and laboratory, were correlated with the progression of diabetic patients with COVID-19, few studies have put these risk factors into a risk score to predict the severity of diabetic patients with COVID-19. Based on our previous data, we took dyspnea, cardiovascular diseases, neutrophil, PLT count, and CKMB as the scoring indicators, and conducted two classification calculation to obtain the weight coefficient (Figure 4). For the convenience of calculation, we expand the weight coefficient of each index by 10 times, an integer value for each risk factor was used to calculate a total score capable of quantifying the risk of severity progression in diabetic patients, that is, DPCR score (Table 4). Dyspnea, cardiovascular disease, PLT count and CKMB each earned a patient 2 points, whereas the neutrophil was worth 3 points in diabetic patients. The maximum score was 11.

**Table 4:** Establishment of risk score of diabetic patients with COVID-19 (DPCR score).

	**Weighted beta coefficient**	**Score**
		
Dyspnea (yes)	0.233	2
Cardiovascular disease (yes)	0.168	2
Neutrophil (>3.85 10^∧^9/L)	0.261	3
PLT ( ≤ 200 10^∧^9/L)	0.199	2
CKMB (>11.2 ng/ml)	0.225	2

**Figure 5:**
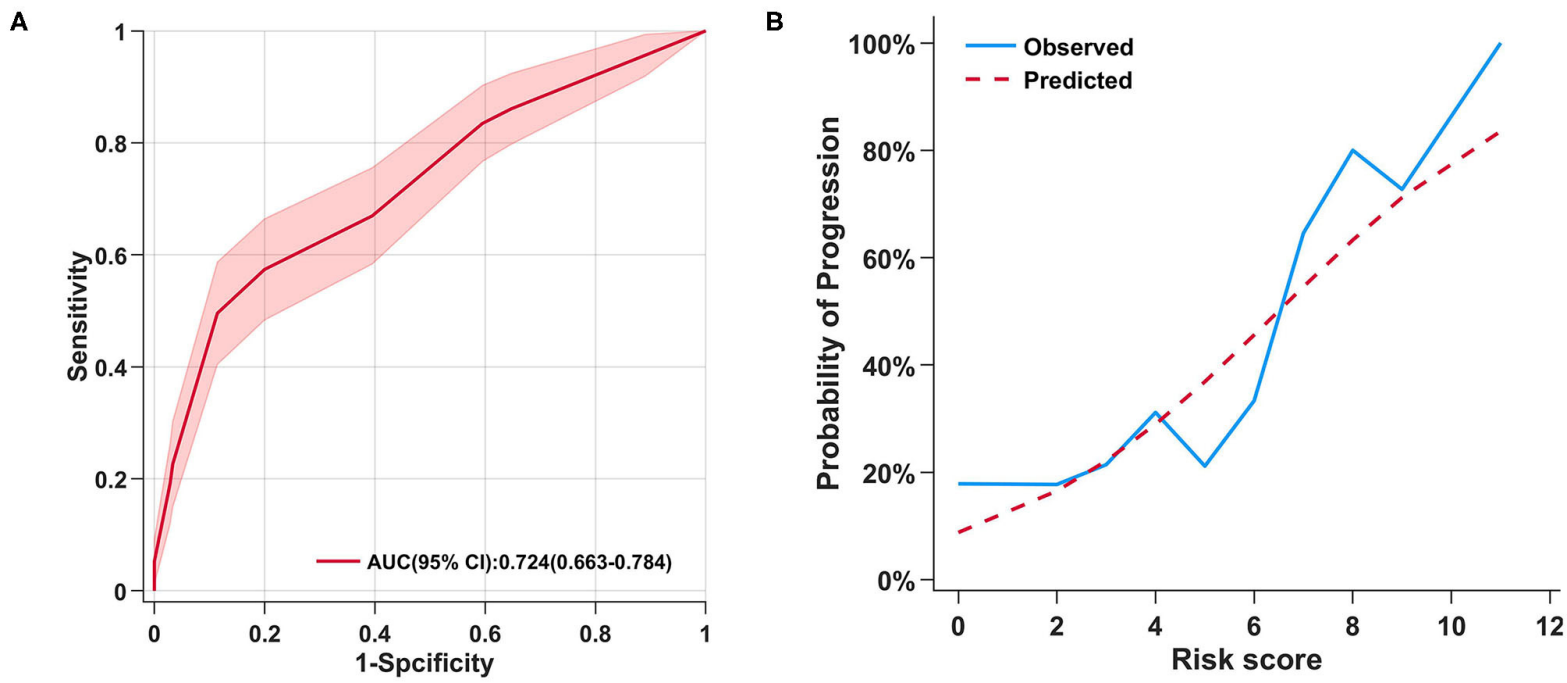
**(A)** ROC curve for risk score as a predictor of severity of diabetic patients with COVID-19; **(B)** Observed vs. predicted probabilities of progression by risk score.

Then, the model's ability to accurately differentiate the risk of progression in diabetic patients with COVID-19 was tested. The area under the receiver operator curve (ROC) was 0.724 (Figure 5A). Predicted and observed rates of progression for each risk score were also compared (Supplementary Table 5). In addition, the Hosmer-Lemeshow test resulted in a *p*-value of 0.194, suggesting that there was no statistical difference between the predicted and observed rates of progression. In addition, a calibration plot was also applied in Figure 5B. Based on the ROC curve, an optimal risk score cutoff of 7 or higher was associated with a high risk of severity progression in diabetic patients with COVID-19. If a diabetic patient had a score of 4 or lower, they were considered to have a low risk of severity progression. The sensitivity and specificity of the model were 0.496 and 0.886, respectively.

## Discussion

Recent studies have shown that patients infected with COVID-19 who also have underlying chronic diseases (7, 8), such as hypertension, cancer, cardiovascular diseases and diabetes, have a higher risk of severe disease and death. Among these chronic diseases, diabetes stood out, not only because it was reported to be the second most common comorbidity of COVID-19, but also because it had been proven by many other studies that diabetes contributes to the risk of mortality following COVID-19 infection.

The potential mechanism by which COVID-19 infection increases the susceptibility and mortality of diabetic patients has also been discussed by many researchers. Muniyappa et al. (9) suspected that higher affinity cellular binding and efficient virus entry, decreased viral clearance, diminished T cell function, increased susceptibility to hyperinflammation and cytokine storm syndrome, and the presence of cardiovascular disease were possible mechanisms leading to the increased severity observed among diabetic patients with COVID-19 (9). Similarly, recent studies showed that angiotensin-converting enzyme 2 (ACE2), transmembrane serine protease 2 (TMPRSS2), sialic acid receptors, matrix metalloproteinase inducer (CD147), catepsin B and L were reported as potential key entry factors in the pathogenesis of COVID-19 (10). ACE2 is also highly expressed in pancreatic beta cells. In addition, previous studies also showed that the state and function of immune cells in diabetic patients were different from those in healthy persons (11, 12). Moreover, senescent T cells showed strong similarities to those in patients with hyperglycemia, and the accumulation of highly differentiated end-stage memory T cells was also found in these patients, which has a detrimental impact on immune function in diabetes (13). T cell senescence in turn contributes to abnormal glucose homeostasis (14), causing a vicious cycle. The above studies demonstrated that the decrease in T cell function laid the foundation for the difference in the immune environment in diabetic patients infected with COVID-19. In our study, we found that the absolute lymphocyte count in diabetic patients was significantly lower than that of normal patients (*p* < 0.001, OR=4.264), suggesting that the decrease in lymphocytes in diabetic patients is related to poor prognosis.

We also found that the absolute value of neutrophils was significantly higher in diabetic patients, which also suggested that the inflammatory response in diabetic patients was significantly higher than that in non-diabetic patients. Recently, Chen et al. found that CRP may help to identify patients with diabetes who were at greater risk of dying during hospitalization (6). They found that high CRP (OR = 1.16, *p* = 0.033) and low albumin (OR = 0.91, *p* = 0.030) were risk factors for poor prognosis in patients with diabetes and COVID-19. Similarly, in our study, we found that CRP was highly expressed in diabetic patients. In addition, the level of CRP was much higher in the DG of diabetic patients than in the SurG of diabetic patients [57.79 mg/L (7.14–169.28) vs. 4.295 mg/L (1.1675–15.7975)]. The high level may be related to the immune function and response of diabetic patients. However, further binary logistic regression analysis revealed that CRP could not be used as a risk factor related to the severity and risk of death of diabetic patients with COVID-19, although the OR value was relatively high. Therefore, we believe that CRP has a certain suggestive role, but it is not significantly related to the severity and death of diabetic patients infected with COVID-19. Conversely, through binary regression analysis, we found that CRP could be a relevant factor for severity and mortality risk in non-diabetic patients.

The level of CKMB increases after myocardial necrosis, which has made it the gold standard for the diagnosis of acute myocardial infarction for many years (15, 16). However, elevations of CKMB were never intended to be synonymous with myocardial infarction, only indicative of cardiac injury, because the expression of CKMB in other tissues impairs specificity (15). A recent study showed that an increase in CKMB, along with other cardiac-specific biomarkers (such as CK, myoglobin, troponin, and NT-proBNP), can play a crucial role in identifying patients vulnerable to developing cardiovascular manifestations of COVID-19(17). Interestingly, we found that the level of CKMB was much higher in diabetic patients than in non-diabetic patients [8.89 ng/ml (6.63–11.42) vs. 9.6 ng/ml (7.6–13.95), *p* < 0.0001, OR = 4.808]. In addition, binary logistic regression analysis also showed that a high level of CKMB was related to the risk of death and disease severity in diabetic patients. The increase in CKMB may be related to hyperglycemia in diabetic patients (18). Qiu et al. Demonstrated, in a diabetic model, that hyperglycemia could induce NLRP3 inflammasome activation, which may lead to pyroptosis and aggravated myocardial ischemia/reperfusion injury. This basic study demonstrated the importance of glycemic control in diabetic patients with COVID-19.

Recently, studies have shown that diabetes is a risk factor for the progression and prognosis of COVID-19, and many laboratory and clinical data have been analyzed. Some indicators have been considered potential targets related to the prognosis of diabetic patients with COVID-19. However, no risk score has been applied to predict the severity of diabetic patients with COVID-19. Here, based on our data, independent risk factors associated with the severity of diabetes mellitus were determined by binary logistic regression analysis: dyspnea, cardiovascular disease, neutrophil, PLT count and CKMB. The risk score was built according to these five factors, which may strongly relate to the severity of disease in diabetic patients with COVID-19. In our risk score, all the data were available to the clinician immediately upon admission to the hospital. In addition, predicted and observed rates of progression for each risk score were also compared.

## Data Availability Statement

The original contributions presented in the study are included in the article/Supplementary Material, further inquiries can be directed to the corresponding author/s.

## Ethics Statement

The studies involving human participants were reviewed and approved by the ethics committee of Xinqiao Hospital (2020-yd073-01) with written informed consent waived due to the retrospective nature of the study. This study was carried out according to the Strengthening the Reporting of Observational Studies in Epidemiology (STROBE) reporting guidelines. The patients/participants provided their written informed consent to participate in this study.

## Author Contributions

Y-FX, J-LH, and YX interpreted results and drafted the manuscript. XL, QL, ZX, M-DH, X-BR, CZ, W-JZ, WD, Y-FT, PL, and HW collected the data. HL analyzed the data. EL built and evaluated the risk score. C-PS and S-MY conceived the study, interpreted results and supervised research. All authors contributed to the article and approved the submitted version.

## Conflict of Interest

The authors declare that the research was conducted in the absence of any commercial or financial relationships that could be construed as a potential conflict of interest.

## Abbreviations

COVID-19: coronavirus disease 19
PLT: platelet
CKMB: creatine kinase-MB
IQR: interquartile range
WBC: white blood cell
CRP: C-reactive protein
MG: mild group
SG: severe group
SurG: survival group
DG: deceased group
ACE2: angiotensin-converting enzyme 2
TMPRSS2: transmembrane serine protease 2.
