# Supplementary material for: Major Characteristics of Severity and Mortality in Diabetic Patients With COVID-19 and Establishment of Severity Risk Score
Source: Front Med (Lausanne). 2021 Jun 7;8:655604. doi: 10.3389/fmed.2021.655604 (PMC8215148; doi:10.3389/fmed.2021.655604)
Supplement: Supplementary file 1 [file Table_1.DOCX]

Sup Table 1 Univariate analysis of severity-related factors in non-diabetic patients with COVID-19 (N=3298)

|  | Total  (N=3298) | Mild  (N=2515) | Severe  (N=783) | *p* value |
| --- | --- | --- | --- | --- |
| Male, n (%) | 1589(48.18) | 1193(47.44) | 396(50.57) | 0.125 |
| Female, n (%) | 1709(51.82) | 1322(52.56) | 387(49.43) | 0.125 |
| Age, median (IQR) | 61(49~69) | 58(47~67) | 67(60~75) | 0.000 |
| Body Temperature, Mean±SD, ℃ | 37.61±1.04 | 37.61±1.02 | 37.64±1.09 | 0.472 |
| Fever, n (%) | 2099(63.64) | 1619(64.37) | 480(61.30) | 0.119 |
| Respiratory rate, n/min | 20(18~21) | 20(18~20) | 20(19~23) | 0.000 |
| Pulse, n/min | 84(78~95) | 84(77~94) | 87(78~99) | 0.000 |
| SBP, median (IQR), mmHg | 130(120~140) | 129(120~139) | 131(120~145) | 0.000 |
| DBP, median (IQR), mmHg | 80(73~88) | 80(73~88) | 80(73~89) | 0.316 |
| Fatigue, n (%) | 1620(49.12) | 1208(48.03) | 412(52.62) | 0.025 |
| Duration of first symptom, day (IQR) | 21(14~30) | 20(13~30) | 25(14~30) | 0.000 |
| Respiratory symptoms |  |  |  |  |
| Cough, n (%) | 2125(64.43) | 1605(63.82) | 520(66.41) | 0.185 |
| Expectoration, n (%) | 404(12.25) | 285(11.33) | 119(15.2) | 0.004 |
| Dyspnea, n (%) | 780(23.65) | 512(20.36) | 268(34.23) | 0.000 |
| Chest tightness, n (%) | 665(20.16) | 474(18.85) | 191(24.39) | 0.001 |
| Hemoptysis, n (%) | 8(0.24) | 6(0.24) | 2(0.26) | 1.000 |
| Digestive tract symptoms |  |  |  |  |
| Vomiting, n (%) | 77(2.33) | 60(2.39) | 17(2.17) | 0.728 |
| Abdominal pain, n (%) | 41(1.24) | 27(1.07) | 14(1.79) | 0.115 |
| Diarrhea, n (%) | 173(5.25) | 141(5.61) | 32(4.09) | 0.096 |
| Anorexia, n (%) | 853(25.86) | 634(25.21) | 219(27.97) | 0.123 |
| Past medical history |  |  |  |  |
| Cardiovascular disease ^*^, n (%) | 776(23.53) | 538(21.39) | 238(30.40) | 0.000 |
| Lung diseases ^†^, n (%) | 147(4.46) | 97(3.86) | 50(6.39) | 0.003 |
| Liver disease ^‡^, n (%) | 99(3.00) | 71(2.82) | 28(3.58) | 0.281 |
| WBC, median (IQR), 10^9/L | 5.80(4.80~6.98) | 5.70(4.73~6.80) | 6.11(4.90~7.80) | 0.000 |
| Neutrophil, median (IQR),10^9/L | 3.45(2.66~4.52) | 3.37(2.60~4.31) | 3.81(2.90~5.38) | 0.000 |
| Lymphocyte, median (IQR), 10^9/L | 1.53(1.14~1.89) | 1.59(1.21~1.94) | 1.32(0.86~1.71) | 0.000 |
| Proportion of neutral lymph, median (IQR) | 2.26(1.63~3.26) | 2.16(1.57~2.94) | 2.74(1.85~5.15) | 0.000 |
| HGB, median (IQR), g/L | 122(111~133) | 123(114~134) | 116(103~128) | 0.000 |
| PLT, median (IQR), 10^9/L | 226(184~272) | 227(187~273) | 221(171~270) | 0.001 |
| Bilirubin, median (IQR), umol/L | 10.30(7.90~13.25) | 1.46(0.50~6.42) | 4.38(0.93~19.70) | 0.000 |
| ALT, median (IQR), IU/L | 24.70(15.20~37.83) | 10.11(7.75~13.25) | 10.90(8.39~13.83) | 0.057 |
| AST, median (IQR), IU/L | 23.10(17.30~37.35) | 24.90(15.50~38.50) | 24.30(14.30~36.30) | 0.293 |
| ALB, median (IQR), g/L | 37.85(34.84~40.30) | 23.00(17.27~37.35) | 23.42(17.40~37.35) | 0.000 |
| CRP, median (IQR), mg/L | 1.81(0.50~6.42) | 38.10(35.70~40.70) | 36.00(32.16~38.82) | 0.000 |
| CREA, median (IQR), umol/L | 62.10(51.58~75.23) | 62.50(52.30~75.00) | 60.31(50.30~77.31) | 0.194 |
| CKMB, median (IQR), ng/mL | 8.89(6.63~11.42) | 8.80(6.63~11.09) | 9.1(6.7~13.3) | 0.013 |
| MuLBSTA Score, median (IQR) | 7(5~9) | 6(5~8) | 9(7~11) | 0.000 |
| Death, n (%) | 79(2.40) | 20(0.80) | 59(7.54) | 0.000 |
| ^*^ Cardiovascular disease includes coronary heart disease and hypertension and etc.  ^†^ Lung disease includes chronic bronchitis, COPD, tuberculosis and lung cancer and etc.  ^‡^ Liver disease includes hepatitis B, hepatitis C, fatty liver, cirrhosis, liver cancer, hepatitis A, hepatic hemangioma, schistosomiasis liver disease and etc. | | | | |
