# Supplementary material for: Major Characteristics of Severity and Mortality in Diabetic Patients With COVID-19 and Establishment of Severity Risk Score
Source: Front Med (Lausanne). 2021 Jun 7;8:655604. doi: 10.3389/fmed.2021.655604 (PMC8215148; doi:10.3389/fmed.2021.655604)
Supplement: Supplementary file 2 [file Table_2.DOCX]

Sup Table 2 Binary logistic regression analysis of severity-related factors in non-diabetic patients with COVID-19

|  | B | SE | Wals | *p* value | OR (95% CI) |
| --- | --- | --- | --- | --- | --- |
|  |  |  |  |  |  |
| Gender | 0.218 | 0.093 | 5.473 | 0.019 | 1.244(1.036, 1.493) |
| Age | 0.039 | 0.004 | 112.958 | 0.000 | 1.040(1.032, 1.047) |
| Respiratory Rate | 0.027 | 0.006 | 20.317 | 0.000 | 1.028(1.016, 1.040) |
| Dyspnea | 0.561 | 0.098 | 32.617 | 0.000 | 1.752(1.446, 2.125) |
| Cardiovascular disease | 0.261 | 0.100 | 6.818 | 0.009 | 1.298(1.067, 1.579) |
| WBC | 0.036 | 0.016 | 5.246 | 0.022 | 1.037(1.005, 1.070) |
| HGB | -0.013 | 0.002 | 30.317 | 0.000 | 0.987(0.982, 0.991) |
| ALB | -0.035 | 0.010 | 11.197 | 0.001 | 0.966(0.946, 0.986) |
| CRP | 0.007 | 0.002 | 15.229 | 0.000 | 1.007(1.003, 1.010) |
| Intercept | 2.107 | 0.574 | 13.479 | 0.000 | 0.122 |
