# Supplementary material for: Major Characteristics of Severity and Mortality in Diabetic Patients With COVID-19 and Establishment of Severity Risk Score
Source: Front Med (Lausanne). 2021 Jun 7;8:655604. doi: 10.3389/fmed.2021.655604 (PMC8215148; doi:10.3389/fmed.2021.655604)
Supplement: Supplementary file 3 [file Table_3.DOCX]

Sup Table 3 Univariate analysis of death-related factors in non-diabetic patients with COVID-19(N=3298)

|  | Total  (N=3298) | Survival (n=3219) | Death  (n=79) | *p* Value |
| --- | --- | --- | --- | --- |
| Male, n (%) | 1589(56.49) | 1540(47.84) | 49(62.03) | 0.013 |
| Female, n (%) | 1709(43.51) | 1679(52.16) | 30(37.97) | 0.013 |
| Age, median (IQR) | 61(49~69) | 60(49~68) | 70(63~82) | 0.000 |
| Body Temperature, Mean±SD, ℃ | 37.61±1.01 | 37.60±1.03 | 37.60±1.15 | 0.367 |
| Fever, n (%) | 2099(53.44) | 2050(63.68) | 49(62.03) | 0.762 |
| Respiratory rate, n/min | 20(18~21) | 20(18~21) | 44(20~26) | 0.000 |
| Pulse, n/min | 84(78~95) | 84(78~95) | 187(80~101) | 0.001 |
| SBP, median (IQR), mmHg | 130(120~140) | 130(120~140) | 262(111~149) | 0.442 |
| DBP, median (IQR), mmHg | 80(73~88) | 80(73~88) | 163(65~88) | 0.073 |
| Fatigue, n (%) | 1620(41.24) | 1579(49.05) | 41(51.90) | 0.170 |
| Duration of first symptom, day (IQR) | 21(14-30) | 21(14~30) | 15(10~24.9) | 0.000 |
| Respiratory symptoms |  |  |  |  |
| Cough, n (%) | 2125(54.10) | 2075(64.46) | 50(63.29) | 0.830 |
| Expectoration, n (%) | 404(10.29) | 394(12.24) | 10(12.66) | 0.911 |
| Dyspnea, n (%) | 780(19.86) | 739(22.96) | 41(51.90) | 0.000 |
| Chest tightness, n (%) | 665(16.93) | 644(20.01) | 21(26.58) | 0.150 |
| Hemoptysis, n (%) | 8(0.20) | 7(0.22) | 1(1.27) | 0.176 |
| Digestive tract symptoms |  |  |  |  |
| Vomiting, n (%) | 77(1.96) | 72(2.24) | 5(6.33) | 0.017 |
| Abdominal pain, n (%) | 42(1.07) | 40(1.24) | 1(1.27) | 1.000 |
| Diarrhea, n (%) | 173(4.40) | 169(5.25) | 4(5.06) | 1.000 |
| Anorexia, n (%) | 853(21.72) | 819(25.44) | 34(43.04) | 0.000 |
| Past medical history |  |  |  |  |
| Cardiovascular disease ^*^, n (%) | 776(19.76) | 755(23.45) | 21(26.58) | 0.517 |
| Lung diseases ^†^, n (%) | 147(3.74) | 136(4.22) | 11(13.92) | 0.000 |
| Liver disease ^‡^, n (%) | 99(2.52) | 97(3.01) | 2(2.53) | 1.000 |
| WBC, median (IQR), 10^9/L | 5.8(4.80~6.98) | 5.74(4.78~6.9) | 7.3(5.40~13.70) | 0.000 |
| Neutrophil, median (IQR),10^9/L | 3.45(2.66~4.52) | 3.43(2.65~4.45) | 5.81(3.83~12.48) | 0.000 |
| Lymphocyte, median (IQR), 10^9/L | 1.53(1.14~1.89) | 1.54(1.16~1.90) | 0.81(0.53~1.47) | 0.000 |
| Proportion of neutral lymph, median (IQR) | 2.26 (1.63~3.26) | 2.24(1.62~3.20) | 8.21(2.63~16.73) | 0.000 |
| HGB, median (IQR), g/L | 122(111~133) | 122(112~133) | 119(106~131) | 0.078 |
| PLT, median (IQR), 10^9/L | 226(184~272) | 227(185~273) | 191(98~254) | 0.000 |
| Bilirubin, median (IQR), umol/L | 10.30(7.90~13.25) | 10.30(7.89~13.25) | 13.00(9.60~19.99) | 0.000 |
| ALT, median (IQR), IU/L | 24.70(15.20~37.83) | 24.70(15.20~37.70) | 26.20(16.10~43.90) | 0.532 |
| AST, median (IQR), IU/L | 23.10(17.30~37.35) | 23.00(17.30~37.35) | 30.70(18.67~38.70) | 0.002 |
| ALB, median (IQR), g/L | 37.85(34.84~40.30) | 37.85(35.10~40.40) | 31.60(27.50~35.40) | 0.000 |
| CRP, median (IQR), mg/L | 1.81(0.50~6.42) | 1.71(0.50~6.42) | 38.49(9.52~103.60) | 0.000 |
| CREA, median (IQR), umol/L | 62.10(51.58~75.23) | 61.80(51.50~75.00) | 72.00(54.83~89.68) | 0.001 |
| CKMB, median (IQR), ng/mL | 8.89(6.63~11.42) | 8.81(6.63~11.31) | 12.80(8.04~18.00) | 0.000 |
| MuLBSTA Score, median (IQR) | 7(5~9) | 7(5~9) | 11(7~15) | 0.000 |
| Diagnosis type |  |  |  |  |
| Mild and Common, n (%) | 2515(64.03) | 2495(77.51) | 20(25.32) | 0.000 |
| Severe and Critical, n (%) | 783(19.93) | 724(22.49) | 59(74.68) |  |
| ^*^ Cardiovascular disease includes coronary heart disease and hypertension and etc.  ^†^ Lung disease includes chronic bronchitis, COPD, tuberculosis and lung cancer and etc.  ^‡^ Liver disease includes hepatitis B, hepatitis C, fatty liver, cirrhosis, liver cancer, hepatitis A, hepatic hemangioma, schistosomiasis liver disease and etc. | | | | |
