# Supplementary material for: Major Characteristics of Severity and Mortality in Diabetic Patients With COVID-19 and Establishment of Severity Risk Score
Source: Front Med (Lausanne). 2021 Jun 7;8:655604. doi: 10.3389/fmed.2021.655604 (PMC8215148; doi:10.3389/fmed.2021.655604)
Supplement: Supplementary file 4 [file Table_4.DOCX]

Sup Table 4 Binary logistic regression analysis of death-related factors in non-diabetic patients with COVID-19

|  | B | SE | Wals | *p* value | OR (95% CI) |
| --- | --- | --- | --- | --- | --- |
|  |  |  |  |  |  |
| Age | 0.036 | 0.012 | 9.688 | 0.002 | 1.037(1.013, 1.060) |
| Dyspnea | 0.853 | 0.273 | 9.750 | 0.002 | 2.347(1.374, 4.011) |
| WBC | 0.048 | 0.014 | 11.797 | 0.001 | 1.049(1.021, 1.078) |
| Neutrophil | 0.050 | 0.014 | 12.129 | 0.000 | 1.051(1.022, 1.081) |
| PLT | -0.006 | 0.002 | 11.596 | 0.001 | 0.994(0.991, 0.998) |
| ALB | -0.091 | 0.025 | 13.499 | 0.000 | 0.913(0.870, 0.959) |
| CRP | 0.015 | 0.003 | 31.955 | 0.000 | 1.015(1.010, 1.020) |
| Severity | 1.350 | 0.327 | 17.096 | 0.000 | 3.859(2.035, 7.320) |
| Intercept | -3.796 | 1.311 | 8.381 | 0.004 | 0.022 |
