# Supplementary material for: Major Characteristics of Severity and Mortality in Diabetic Patients With COVID-19 and Establishment of Severity Risk Score
Source: Front Med (Lausanne). 2021 Jun 7;8:655604. doi: 10.3389/fmed.2021.655604 (PMC8215148; doi:10.3389/fmed.2021.655604)
Supplement: Supplementary file 5 [file Table_5.DOCX]

Sup Table 5 Predicted and observed rates of progression for each risk score in DPCR score

|  | Observed Progression | Predicted Progression | AUC (95% CI) |
| --- | --- | --- | --- |
| Risk score |  |  | 0.724(0.663-0.784) |
| 0 | 17.86% | 8.79% |  |
| 2 | 17.74% | 16.54% |  |
| 3 | 21.43% | 22.13% |  |
| 4 | 31.15% | 28.95% |  |
| 5 | 21.15% | 36.88% |  |
| 6 | 33.33% | 45.59% |  |
| 7 | 64.58% | 54.58% |  |
| 8 | 80.00% | 63.28% |  |
| 9 | 72.73% | 71.19% |  |
| 11 | 100.00% | 83.56% |  |
| Hosmer-Lemeshow | χ^2^ =11.144 | P=0.194 |  |
| Regression model |  |  | 0.725(0.666-0.785) |
|  | Odds ratio | 95% CI | P |
| Risk ≥ 7 | 7.616 | 4.348-13.343 | <0.001 |
